# Catastrophic health expenditure in sub-Saharan Africa: systematic review and meta-analysis

**DOI:** 10.2471/BLT.21.287673

**Published:** 2022-04-04

**Authors:** Paul Eze, Lucky Osaheni Lawani, Ujunwa Justina Agu, Yubraj Acharya

**Affiliations:** a Department of Health Policy and Administration, 504A Donald H. Ford Building, Pennsylvania State University, University Park, Pennsylvania, PA 16802, United States of America.; b Institute of Health Policy, Management & Evaluation, University of Toronto, Toronto, Canada.; c Department of Community Medicine, Enugu State University Teaching Hospital, Parklane, Nigeria.

## Abstract

**Objective:**

To estimate the incidence of, and trends in, catastrophic health expenditure in sub-Saharan Africa.

**Methods:**

We systematically reviewed the scientific and grey literature to identify population-based studies on catastrophic health expenditure in sub-Saharan Africa published between 2000 and 2021. We performed a meta-analysis using two definitions of catastrophic health expenditure: 10% of total household expenditure and 40% of household non-food expenditure. The results of individual studies were pooled by pairwise meta-analysis using the random-effects model.

**Findings:**

We identified 111 publications covering a total of 1 040 620 households across 31 sub-Saharan African countries. Overall, the pooled annual incidence of catastrophic health expenditure was 16.5% (95% confidence interval, CI: 12.9–20.4; 50 datapoints; 462 151 households; *I*^2^ = 99.9%) for a threshold of 10% of total household expenditure and 8.7% (95% CI: 7.2–10.3; 84 datapoints; 795 355 households; *I*^2^ = 99.8%) for a threshold of 40% of household non-food expenditure. Countries in central and southern sub-Saharan Africa had the highest and lowest incidence, respectively. A trend analysis found that, after initially declining in the 2000s, the incidence of catastrophic health expenditure in sub-Saharan Africa increased between 2010 and 2020. The incidence among people affected by specific diseases, such as noncommunicable diseases, HIV/AIDS and tuberculosis, was generally higher.

**Conclusion:**

Although data on catastrophic health expenditure for some countries were sparse, the data available suggest that a non-negligible share of households in sub-Saharan Africa experienced catastrophic expenditure when accessing health-care services. Stronger financial protection measures are needed.

## Résumé

**Objectif:**

Évaluer le taux d'incidence et les tendances liées aux dépenses de santé catastrophiques en Afrique subsaharienne.

**Méthodes:**

Nous avons procédé à une revue systématique de la littérature scientifique et de la littérature grise afin d'identifier les études relatives aux dépenses de santé catastrophiques, menées auprès des populations d'Afrique subsaharienne entre 2000 et 2021. Nous avons ensuite effectué une méta-analyse en définissant ces dépenses de deux manières: 10% des dépenses totales d'un ménage et 40% de ses dépenses non alimentaires. Les résultats de chaque étude ont été regroupés par paires de méta-analyses à l'aide d'un modèle à effets aléatoires.

**Résultats:**

Nous avons sélectionné 111 publications représentant un total de 1 040 620 ménages répartis dans 31 pays d'Afrique subsaharienne. Globalement, le taux d'incidence annuel combiné des dépenses de santé catastrophiques s'élevait à 16,5% (intervalle de confiance de 95%, IC: 12,9–20,4; 50 points de données; 462 151 ménages; *I*^2^ = 99,9%) pour un seuil de 10% des dépenses totales d'un ménage, et à 8,7% (IC de 95%: 7,2–10,3; 84 points de données; 795 355 ménages; *I*^2^ = 99,8%) pour un seuil de 40% de ses dépenses non alimentaires. Les pays situés au centre de l'Afrique subsaharienne présentaient le taux d'incidence le plus élevé, tandis que ceux situés dans la région méridionale affichaient le taux d'incidence le moins élevé. Une analyse des tendances a permis de découvrir qu'après une baisse initiale survenue durant les années 2000, le taux d'incidence des dépenses de santé catastrophiques a augmenté entre 2010 et 2020 en Afrique subsaharienne. En outre, chez les personnes souffrant de pathologies spécifiques comme les maladies non transmissibles, le VIH/SIDA ou la tuberculose, ce taux d'incidence était généralement plus élevé.

**Conclusion:**

Malgré le manque de données disponibles à ce propos dans certains pays, les informations en notre possession semblent indiquer qu'une part non négligeable des ménages d'Afrique subsaharienne est confrontée à des dépenses catastrophiques en matière d'accès aux soins de santé. Des mesures de protection financière renforcées sont donc nécessaires.

## Resumen

**Objetivo:**

Estimar la tasa de incidencia y las tendencias de los gastos sanitarios catastróficos en el África subsahariana.

**Métodos:**

Se revisó sistemáticamente la literatura científica y gris para identificar los estudios poblacionales sobre el gasto sanitario catastrófico en el África subsahariana que fueron publicados entre 2000 y 2021. Se realizó un metanálisis empleando dos definiciones de gasto sanitario catastrófico: 10 % del gasto total del hogar y 40 % del gasto no alimentario del hogar. Los resultados de los estudios individuales se agruparon mediante un metanálisis por pares utilizando el modelo de efectos aleatorios.

**Resultados:**

Se identificaron 111 publicaciones que incluían un total de 1 040 620 hogares en 31 países del África subsahariana. En general, la tasa de incidencia anual conjunta de los gastos sanitarios catastróficos fue del 16,5 % (intervalo de confianza del 95 %, IC: 12,9-20,4; 50 observaciones estadísticas; 462 151 hogares; *I*^2^ = 99,9 %) para un umbral del 10 % del gasto total del hogar y del 8,7 % (IC del 95 %: 7,2-10,3; 84 observaciones estadísticas; 795 355 hogares; *I*^2^ = 99,8 %) para un umbral del 40 % del gasto no alimentario del hogar. Los países del centro y del sur del África subsahariana presentaron la mayor y la menor tasa de incidencia, respectivamente. Un análisis de la tendencia reveló que, tras disminuir inicialmente en la década de 2000, la tasa de incidencia de los gastos sanitarios catastróficos en el África subsahariana aumentó entre 2010 y 2020. La tasa de incidencia entre las personas afectadas por enfermedades específicas, como las enfermedades no transmisibles, el VIH/SIDA y la tuberculosis, fue en general mayor.

**Conclusión:**

Aunque los datos sobre los gastos sanitarios catastróficos de algunos países fueron escasos, los datos disponibles sugieren que una parte significativa de los hogares del África subsahariana sufrieron gastos catastróficos al acceder a los servicios sanitarios. Por lo tanto, se requieren medidas de protección financiera más sólidas.

## ملخص

**الغرض:**

تقدير حدوث واتجاهات الإنفاق الصحي الكارثي في جنوب الصحراء الكبرى بأفريقيا.

**الطريقة:**

قمنا بمراجعة منهجية للمنشورات العلمية وغير الرسمية لتحديد الدراسات السكانية حول الإنفاق الصحي الكارثي في جنوب الصحراء الكبرى بأفريقيا، والتي نُشرت بين عامي 2000 و2021. قم بإجراء تحليل تلوي باستخدام تعريفين للإنفاق الصحي الكارثي: 10% من إجمالي الإنفاق الأسري، و40% من نفقات الأسرة غير الغذائية. تم تجميع نتائج الدراسات الفردية عن طريق التحليل التلوي الزوجي باستخدام نموذج التأثيرات العشوائية.

**النتائج:**

قمنا بتحديد 111 مطبوعة تغطي إجمالي 1040620 أسرة في 31 دولة في جنوب الصحراء الكبرى بأفريقيا. بشكل عام، كان الحدوث السنوي المجمع للإنفاق الصحي الكارثي 16.5% (بفاصل ثقة مقداره 95%: 12.9 إلى 20.4؛ 50 نقطة بيانات؛ *I *^2 ^ = 99.9%) لعتبة تبلغ 10% من إجمالي الإنفاق الأسري، و8.7% (بفاصل ثقة مقداره 95%: 7.2 إلى 10.3؛ 84 نقطة بيانات؛ 795355 أسرة؛ *I *^2 ^ = 99.8%) لعتبة تبلغ 40% من إجمالي الإنفاق الأسري غير الغذائي. وسجلت الدول الواقعة في وسط وجنوب الصحراء الكبرى بأفريقيا أعلى وأدنى معدل حدوث على التوالي. اكتشف تحليل للاتجاهات، أنه بعد الانخفاض المبدئي في بداية الألفينات، زاد معدل حدوث الإنفاق الصحي الكارثي في جنوب الصحراء الكبرى بأفريقيا بين عامي 2010 و2020. إن معدل الحدوث بين الأشخاص المصابين بأمراض معينة، مثل الأمراض غير المعدية، وفيروس نقص المناعة البشرية (HIV)/الإيدز (AIDS) والسل، كان أعلى بشكل عام.

**الاستنتاج:**

بالرغم من أن البيانات بخصوص الإنفاق الصحي الكارثي في بعض الدول كانت نادرة، فإن البيانات المتاحة توضح أن نسبة غير قليلة من الأسر في جنوب الصحراء الكبرى بأفريقيا، شهدت إنفاقًا كارثيًا عند الحصول على خدمات الرعاية الصحية. هناك حاجة إلى تدابير للحماية المالية أكثر قوة.

## 摘要

**目的:**

评估撒哈拉以南非洲地区的灾难性医疗支出发生率及趋势

**方法:**

我们系统地评估了科学和灰色文献,以识别 2000 年至 2021 年间发表的关于撒哈拉以南非洲地区灾难性医疗支出的基于人群的研究。我们基于灾难性医疗支出的两个定义开展了元分析:总家庭支出的 10% 和家庭非食品支出的 40%。使用随机效应模型通过成对元分析汇总各个研究的结果。

**结果:**

我们识别了 111 份出版文献,涵盖共 31 个撒哈拉以南非洲国家的 1,040,620 个家庭。总体而言,对于 10% 的家庭总支出阈值,每年的灾难性医疗支出发生率为 16.5%(95% 置信区间,CI:12.9–20.4;50 个数据点;462,151 个家庭;*I*^2^ = 99.9%),对于 40% 的家庭非食品支出阈值,发生率为 8.7%(95% CI:7.2-10.3;84 个数据点;795,355 个家庭;*I*^2^ = 99.8%)。撒哈拉以南中部和南部非洲国家的支出发生率分别最高和最低。一项趋势分析发现,在 2000 年代最初下降之后,撒哈拉以南非洲地区的灾难性医疗支出发生率在 2010 年至 2020 年间有所增加。受非传染性疾病、艾滋病毒/艾滋病和肺结核等特殊疾病影响的人群的灾难性医疗支出发生率普遍较高。

**结论:**

尽管一些国家的灾难性医疗支出数据很少,但现有数据表明,在撒哈拉以南的非洲地区,有一部分家庭在获得卫生保健服务时也支出了灾难性医疗费用。有必要采取更强有力的金融保护措施。

## Резюме

**Цель:**

Оценить масштабы и тенденции катастрофических расходов на здравоохранение в странах Африки, расположенных к югу от Сахары.

**Методы:**

Авторы выполнили систематический обзор научной и «серой» литературы для выявления популяционных исследований о катастрофических расходах на здравоохранение в странах Африки, расположенных к югу от Сахары, которая была опубликована в период с 2000 по 2021 год. Авторы провели метаанализ, используя два определения катастрофических расходов на здравоохранение: 10% от общих расходов домохозяйства и 40% от непродовольственных расходов домохозяйства. Результаты отдельных исследований были объединены с помощью попарного метаанализа с использованием модели случайных эффектов.

**Результаты:**

Авторы обнаружили 111 публикаций, охватывающих в общей сложности 1 040 620 домохозяйств в 31 стране Африки, расположенной к югу от Сахары. В целом совокупная годовая частота катастрофических расходов на здравоохранение составила 16,5% (95%-й ДИ: 12,9–20,4; 50 точек данных; 462 151 домохозяйство;* I*^2^ = 99,9%) для порогового уровня в 10% от общих расходов домохозяйства и 8,7% (95%-й ДИ: 7,2–10,3; 84 точки данных; 795 355 домохозяйств;* I*^2^ = 99,8%) для порогового уровня в 40% от непродовольственных расходов домохозяйства. Страны центральной и южной частей Африки, расположенные к югу от Сахары, имели самый высокий и самый низкий уровень заболеваемости соответственно. Анализ тенденций показал, что после первоначального снижения в 2000-х годах частота катастрофических расходов на здравоохранение в странах Африки, расположенных к югу от Сахары, увеличилась в период с 2010 по 2020 год. Заболеваемость среди людей, страдающих специфическими заболеваниями, такими как неинфекционные заболевания, ВИЧ/СПИД и туберкулез, в целом была выше.

**Вывод:**

Хотя данные о катастрофических расходах на здравоохранение в некоторых странах были достаточно скудными, имеющиеся данные свидетельствуют о том, что значительная часть домохозяйств в странах Африки, расположенных к югу от Сахары, несла катастрофические расходы при доступе к услугам здравоохранения. Поэтому необходимо внедрять более действенные меры финансовой защиты.

## Introduction

In 2019, over 930 million people worldwide experienced financial hardship while obtaining health care and, annually, about 100 million people were impoverished.^1^ Out-of-pocket payments, the predominant form of health care financing in sub-Saharan Africa, have hindered the region’s drive towards universal health coverage (UHC) and attainment of the sustainable development goals (SDGs).^2^^–^^4^ Moreover, payments affect the poorest households disproportionately, thereby exacerbating inequality.^3^^,^^5^

Catastrophic health expenditure has been defined as out-of-pocket payments above a share of total household expenditure or non-food expenditure that forces households to sacrifice other basic needs, sell assets, incur debts or become impoverished.^6^^,^^7^ This perpetuates a vicious cycle of poverty for poor households and leads to more illness when households cannot afford out-of-pocket costs.^2^^,^^8^ Reducing the incidence of catastrophic health expenditure is a key policy objective of governments in sub-Saharan Africa.^2^ However, the design and implementation of appropriate policies requires accurate, up-to-date evidence on the incidence of catastrophic health expenditure, which is scant at present.

Our aim was to fill this evidence gap by performing a systematic review of population-based studies of catastrophic health expenditure in sub-Saharan Africa. In particular, we aimed to estimate the magnitude of, and between-country variation in, the annual incidence of catastrophic health expenditure between 2000 and 2021 and to investigate trends over time.

## Methods

We searched the PubMed®, African Journals Online, CINAHL, CNKI, African Index Medicus, PsycINFO, SciELO, Scopus and Web of Science databases using terms covering catastrophic health expenditure, financial catastrophe and sub-Saharan Africa (Box 1; available at: https://www.who.int/publications/journals/bulletin/) for studies published between 1 January 2000 and 30 September 2021 in the 48 countries of sub-Saharan Africa (Box 2), as defined by the World Bank.^9^ In addition, two authors independently searched the published literature between 2 October and 10 October 2021. We also searched the New York Academy of Medicine Grey Literature and Open Grey, two prepublication server depositories (i.e. medRxIV and bioRxIV) and Google Scholar^®^ for grey literature and followed up citations in studies identified through the database search. We considered studies published in any of the six African Union languages: Arabic, English, French, Kiswahili, Portuguese and Spanish. Studies not in English were translated. The two authors underwent a moderation exercise to ensure that inclusion and exclusion criteria (Box 3) were applied uniformly before independently assessing titles and abstracts. Discrepancies were resolved by discussion. Finally, the full texts of eligible articles were assessed against the inclusion criteria. We registered the study protocol on PROSPERO (CRD42021274830) and findings were reported according to PRISMA guidelines.^11^

Box 1 **Literature search strategy, meta-analysis of catastrophic health expenditure in sub-Saharan Africa, 2000–2021**

Search: (((“catastrophe”[All Fields] OR “catastrophes”[All Fields] OR “catastrophic”[All Fields] OR “catastrophically”[All Fields]) AND (“health expenditures”[MeSH Terms] OR (“health”[All Fields] AND “expenditures”[All Fields]) OR “health expenditures”[All Fields] OR (“health”[All Fields] AND “expenditure”[All Fields]) OR “health expenditure”[All Fields])) OR ((“catastrophe”[All Fields] OR “catastrophes”[All Fields] OR “catastrophic”[All Fields] OR “catastrophically”[All Fields]) AND (“health”[MeSH Terms] OR “health”[All Fields] OR “health s”[All Fields] OR “healthful”[All Fields] OR “healthfulness”[All Fields] OR “healths”[All Fields]) AND (“expense”[All Fields] OR “expenses”[All Fields] OR “expensive”[All Fields] OR “expensively”[All Fields])) OR ((“catastrophe”[All Fields] OR “catastrophes”[All Fields] OR “catastrophic”[All Fields] OR “catastrophically”[All Fields]) AND (“health”[MeSH Terms] OR “health”[All Fields] OR “health s”[All Fields] OR “healthful”[All Fields] OR “healthfulness”[All Fields] OR “healths”[All Fields]) AND “expen*”[All Fields]) OR ((“economical”[All Fields] OR “economics”[MeSH Terms] OR “economics”[All Fields] OR “economic”[All Fields] OR “economically”[All Fields] OR “economics”[MeSH Subheading] OR “economization”[All Fields] OR “economize”[All Fields] OR “economized”[All Fields] OR “economizes”[All Fields] OR “economizing”[All Fields]) AND (“impoverish”[All Fields] OR “impoverished”[All Fields] OR “impoverishes”[All Fields] OR “impoverishing”[All Fields] OR “impoverishment”[All Fields])) OR ((“economics”[MeSH Terms] OR “economics”[All Fields] OR “financial”[All Fields] OR “financially”[All Fields] OR “financials”[All Fields] OR “financier”[All Fields] OR “financiers”[All Fields]) AND (“impoverish”[All Fields] OR “impoverished”[All Fields] OR “impoverishes”[All Fields] OR “impoverishing”[All Fields] OR “impoverishment”[All Fields])) AND (“angola”[MeSH Terms] OR “angola”[All Fields] OR “angola s”[All Fields] OR (“benin”[MeSH Terms] OR “benin”[All Fields] OR “benin s”[All Fields]) OR (“botswana”[MeSH Terms] OR “botswana”[All Fields] OR “botswana s”[All Fields]) OR (“burkina faso”[MeSH Terms] OR (“burkina”[All Fields] AND “faso”[All Fields]) OR “burkina faso”[All Fields]) OR (“burundi”[MeSH Terms] OR “burundi”[All Fields]) OR (“cabo verde”[MeSH Terms] OR (“cabo”[All Fields] AND “verde”[All Fields]) OR “cabo verde”[All Fields]) OR (“cameroon”[MeSH Terms] OR “cameroon”[All Fields] OR “cameroons”[All Fields] OR “cameroon s”[All Fields]) OR (“central african republic”[MeSH Terms] OR (“central”[All Fields] AND “african”[All Fields] AND “republic”[All Fields]) OR “central african republic”[All Fields]) OR (“chad”[MeSH Terms] OR “chad”[All Fields]) OR (“comoros”[MeSH Terms] OR “comoros”[All Fields] OR “comoro”[All Fields]) OR “democratic republic congo”[All Fields] OR “republic congo”[All Fields] OR “Cote d'Ivoire”[All Fields] OR (“equatorial guinea”[MeSH Terms] OR (“equatorial”[All Fields] AND “guinea”[All Fields]) OR “equatorial guinea”[All Fields]) OR (“eritrea”[MeSH Terms] OR “eritrea”[All Fields]) OR (“eswatini”[MeSH Terms] OR “eswatini”[All Fields]) OR (“ethiopia”[MeSH Terms] OR “ethiopia”[All Fields] OR “ethiopia s”[All Fields]) OR (“gabon”[MeSH Terms] OR “gabon”[All Fields]) OR (“gambia”[MeSH Terms] OR “gambia”[All Fields] OR “the gambia”[All Fields]) OR (“ghana”[MeSH Terms] OR “ghana”[All Fields] OR “ghana s”[All Fields]) OR (“guinea”[MeSH Terms] OR “guinea”[All Fields] OR “guinea s”[All Fields] OR “guineas”[All Fields]) OR (“guinea bissau”[MeSH Terms] OR “guinea bissau”[All Fields] OR (“guinea”[All Fields] AND “bissau”[All Fields]) OR “guinea bissau”[All Fields]) OR (“kenya”[MeSH Terms] OR “kenya”[All Fields] OR “kenya s”[All Fields]) OR (“lesotho”[MeSH Terms] OR “lesotho”[All Fields]) OR (“liberia”[MeSH Terms] OR “liberia”[All Fields] OR “liberia s”[All Fields]) OR (“madagascar”[MeSH Terms] OR “madagascar”[All Fields] OR “madagascar s”[All Fields]) OR (“malawi”[MeSH Terms] OR “malawi”[All Fields] OR “malawi s”[All Fields]) OR (“mali”[MeSH Terms] OR “mali”[All Fields]) OR (“mauritania”[MeSH Terms] OR “mauritania”[All Fields]) OR (“mauritius”[MeSH Terms] OR “mauritius”[All Fields]) OR (“mozambique”[MeSH Terms] OR “mozambique”[All Fields] OR “mozambique s”[All Fields]) OR (“namibia”[MeSH Terms] OR “namibia”[All Fields]) OR (“niger”[MeSH Terms] OR “niger”[All Fields]) OR (“nigeria”[MeSH Terms] OR “nigeria”[All Fields] OR “nigeria s”[All Fields]) OR (“rwanda”[MeSH Terms] OR “rwanda”[All Fields] OR “rwanda s”[All Fields]) OR “Sao Tome and Principe”[All Fields] OR (“senegal”[MeSH Terms] OR “senegal”[All Fields] OR “senegal s”[All Fields]) OR (“seychelles”[MeSH Terms] OR “seychelles”[All Fields]) OR “Sierra Leone”[All Fields] OR (“somalia”[MeSH Terms] OR “somalia”[All Fields]) OR “South Africa”[All Fields] OR “South Sudan”[All Fields] OR (“sudan”[MeSH Terms] OR “sudan”[All Fields] OR “sudans”[All Fields] OR “sudan s”[All Fields]) OR (“tanzania”[MeSH Terms] OR “tanzania”[All Fields] OR “tanzania s”[All Fields]) OR (“togo”[MeSH Terms] OR “togo”[All Fields]) OR (“uganda”[MeSH Terms] OR “uganda”[All Fields] OR “uganda s”[All Fields]) OR (“zambia”[MeSH Terms] OR “zambia”[All Fields] OR “zambia s”[All Fields]) OR (“zimbabwe”[MeSH Terms] OR “zimbabwe”[All Fields] OR “zimbabwe s”[All Fields])))

Note: Databases were searched for articles published between 2000 and 2021.

Box 2 **Countries included, meta-analysis of catastrophic health expenditure in sub-Saharan Africa, 2000–2021**

Central Africa: Burundi, Cameroon, Central African Republic, Chad, Congo, Democratic Republic of the Congo, Equatorial Guinea, Gabon and Sao Tome and Principe.

Eastern Africa: Comoros, Djibouti, Eritrea, Ethiopia, Kenya, Madagascar, Mauritius, Rwanda, Seychelles, Somalia, South Sudan, Sudan, Uganda and United Republic of Tanzania.

Southern Africa: Angola, Botswana, Eswatini, Lesotho, Malawi, Mozambique, Namibia, South Africa, Zambia and Zimbabwe.

Western Africa: Benin, Burkina Faso, Cabo Verde, Côte d’Ivoire, Gambia, Ghana, Guinea, Guinea-Bissau, Liberia, Mali, Niger, Nigeria, Senegal, Sierra Leone and Togo.

Notes: The list includes the 48 countries of the sub-Saharan African region, as defined by the World Bank.^9^ Countries were grouped into four regions using the African Union classification.^10^

Box 3 **Study inclusion and exclusion criteria, meta-analysis of catastrophic health expenditure in sub-Saharan Africa, 2000–2021**

**Inclusion criteria**

- Observational or interventional studies (which included data on the pre-intervention period) published between 2000 and 2021 that reported population-level data for any of the 48 sub-Saharan African countries defined by the World Bank (Box 2).^9^
- Studies reported in the published or unpublished (i.e. grey) literature.
- Publications that reported the incidence of catastrophic health expenditure for all individuals of all ages in the community as identified through household surveys or through studies based in health facilities that were representative of the entire community.
- Peer-reviewed publications in Arabic, English, French, Portuguese, Spanish or Kiswahili.
- Publications that estimated catastrophic health expenditure using either total household expenditure or income or non-subsistence expenditure.
- Publications that reported data on catastrophic health expenditure that could be extracted as an independent outcome along with the study population (i.e. the denominator).

**Exclusion criteria**

- Publications that reported the incidence or proportion of catastrophic health expenditure based on a retrospective analysis of patients’ charts, an analysis of hospital or pharmacy revenues, or a national or subnational budget analysis.
- Publications that reported the incidence of catastrophic health expenditure for all individuals of all ages based on studies carried out in one or several health facilities (e.g. outpatient clinics, hospitals with inpatients, intensive care units, operating theatres, nursing homes or long-term care facilities) that were not representative of the entire community.
- Interventional studies that reported the incidence of catastrophic health expenditure only after the intervention.
- Studies that used methods for estimating catastrophic health expenditure that were not clearly reported or defined or that reported catastrophic expenditure using terms such as “excessive out-of-pocket health care” or the multidimensional poverty index.
- Articles that reported data for a population already included in the systematic review.
- Case reports, case series, systematic reviews, narrative reviews, letters to editors, commentary pieces and study protocols.

Three authors independently extracted data from the included studies on: (i) study countries; (ii) year of publication; (iii) study design; (iv) data sources; (v) year of data collection; (vi) study population; (vii) sample size; and (viii) the incidence of catastrophic health expenditure as determined using a threshold of 10% of total household expenditure or 40% of household non-food expenditure or both. For surveys spanning several years, we regarded the survey’s first year as the date of the survey. We grouped countries into four regions (i.e. central, eastern, southern and western Africa) using the African Union classification (Box 2) and into three income categories (i.e. low, lower middle and upper middle) using the World Bank’s classification.^9^^,^^10^ We obtained data on social health insurance programme coverage as a percentage of the country’s population from the World Bank and on the UHC’s service coverage index from the World Health Organization’s (WHO) Global Health Expenditure Database.^12^^,^^13^ The service coverage index for 2015 was used for studies whose data were collected before 2016, whereas the index for 2017 was used for all other studies.^13^

Although studies have used different thresholds to define catastrophic health expenditure,^6^^,^^14^ the two most widely used are 10% of total household expenditure and 40% of household non-food expenditure.^15^^,^^16^ We estimated the annual incidence of catastrophic expenditure from the studies included using these thresholds. If catastrophic expenditure was not reported using either of these two definitions, we contacted the study’s authors for supplementary information. We included catastrophic expenditure estimates based on the medical expenditure incurred only;^14^ estimates based on indirect costs, such as transportation, were excluded. We contacted study authors if estimates were missing or reported only monthly or weekly. If two or more studies used the same secondary data to estimate the incidence of catastrophic health expenditure, we used estimates from peer-reviewed studies and from studies that reported catastrophic health expenditure using both definitions.

Three authors independently assessed study quality using the appraisal tool for cross-sectional studies (AXIS) – a 20-question checklist designed to assess a study’s risk of bias across five domains: introduction, methods, results, discussion and other information.^17^ Each study was scored between 0 and 20, with a high score indicating a low risk of bias. Discrepancies between authors were resolved by discussion.

### Data analysis

We used descriptive statistics to summarize the studies’ characteristics. Individual results were pooled by pairwise meta-analysis using the random-effects model (DerSimonian-Laird approach) and the MetaProp Stata command with the Freeman-Tukey double arcsine transformation.^18^ We conducted separate meta-analyses for the two definitions of catastrophic health expenditure. Between-study heterogeneity was assessed using the *χ^2^* test with Cochran’s *Q* statistic and quantified using the *I*^2^ statistic. We used Stata v. 17.0 (StataCorp LLC, College Station, United States of America) for all statistical analyses and an *α* of 0.05 was the cut-off for statistical significance.

We assessed the sensitivity of the pooled estimates to sample size by excluding the 10% of studies with the smallest sample size and the 10% with the largest sample size. The robustness of the estimates was assessed by excluding: (i) studies with the largest and smallest sample sizes; (ii) studies using pre-intervention data; (iii) low-quality studies; and (iv) studies that were not peer reviewed. We performed subgroup analyses along multiple dimensions, including: (i) the data collection period (i.e. 2000 to 2004, 2005 to 2009, 2010 to 2014 and 2015 to 2019); (ii) region (i.e. eastern, central, southern or western Africa); (iii) the country’s income status (i.e. low, lower middle or upper middle); (iv) data type (i.e. primary or secondary); (v) publication status (i.e. peer-reviewed or not); (vi) UHC service coverage index (dichotomized to < 45 and ≥ 45, based on the sub-Saharan African average reported by WHO);^13^ (vii) the proportion of households with social insurance (i.e. < 10% or ≥ 10%); and (viii) the studies’ risk of bias (i.e. high or low, corresponding to an AXIS score of 0–10 or 11–20, respectively).

Finally, we performed a meta-regression analysis to explore factors associated with between-study heterogeneity for all catastrophic health expenditure incidence estimates pooled from 10 or more datapoints.^19^ To avoid overfitting the model, we included a limited number of covariates (selected on the basis of previous studies). Covariates fell into two categories: (i) study-level factors, namely study design, study period, data type and study quality based on the AXIS score;^15^^,^^16^ and (ii) country-level factors, namely income status, UHC service coverage index and the proportion of the population with social insurance.^2^^,^^4^^,^^7^ We also evaluated evidence of publication bias by examining funnel plot symmetry; we performed Egger’s test for small-study effects and used the trim-and-fill method.^19^

We assessed overall evidence quality using the Grading of Recommendations, Assessment, Development and Evaluation (GRADE) approach.^20^ First, we scored the evidence for each outcome as high and downgraded it by one level if one of the following was present: (i) poor methodological quality (i.e. if 25% or more of the studies in the meta-analysis had a high risk of bias); (ii) imprecision (i.e. if 25% or more of the studies did not have a sample size of at least 385 households – the smallest sample size at the 95% confidence interval [CI] and 5% error margin); (iii) indirectness (i.e. if 25% or more of the studies did not use valid and reliable methods of data collection, such as validated questionnaires that had been trialled, piloted or published previously); and (iv) inconsistency (i.e. if the prediction interval for the outcome had a variation of 10% or more between the upper and lower limits of the 95% CI). These criteria were based on Joanna Briggs guidelines, which correspond to the GRADE system criteria.^21^

## Results

**Fig. 1:**
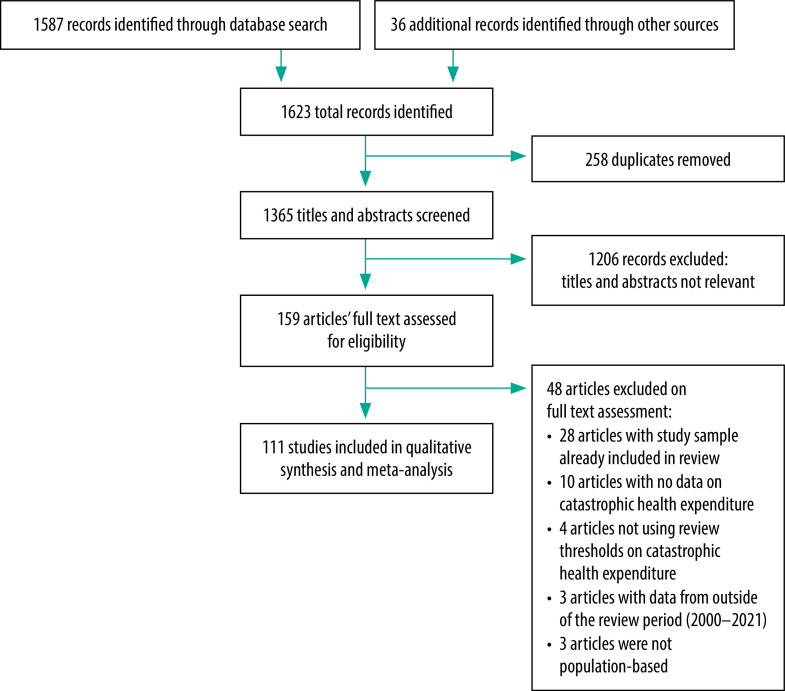
Selection of publications, systematic review of catastrophic health expenditure, sub-Saharan Africa, 2000–2021

**Fig. 2:**
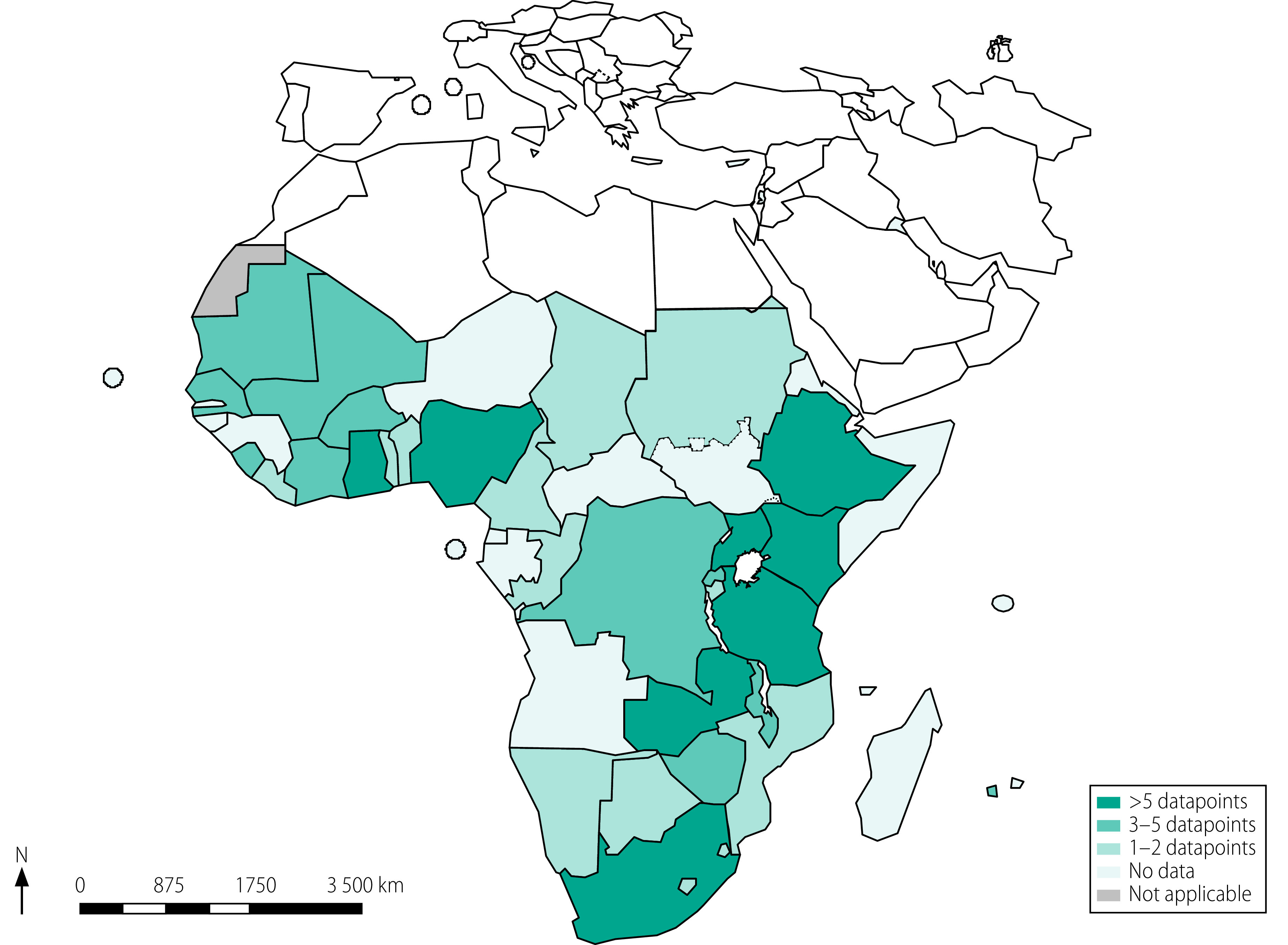
Geographical distribution of studies, meta-analysis of catastrophic health expenditure, sub-Saharan Africa, 2000–2021

Note: The 111 studies identified in the systematic review included 145 datapoints on the annual incidence of catastrophic health expenditure in a specific country in a specific year.

Our initial search identified 1623 studies, including 36 from Google Scholar and citation tracking (Fig. 1). After removing duplicates, 1365 titles and abstracts were screened. Of the 159 articles whose full text was assessed, 111 finally met the inclusion criteria (Table 1; available at: https://www.who.int/publications/journals/bulletin/):^22^^–^^132^ 101 peer-reviewed publications, five working papers, four graduate dissertations and one preprint. Details of the 48 articles excluded are available from the data repository.^133^ All 111 studies were published between 2005 and 2021, 107 (96.4%) were in English and study data were collected between 2000 and 2019. The studies covered a total of 1 040 620 households across 31 countries in sub-Saharan Africa (Fig. 2) and reported 145 distinct datapoints: 50 derived from primary data and 95 derived from secondary data. Each datapoint represented a value for the annual incidence of catastrophic health expenditure in a specific country in a specific year. Of the 145 datapoints, 6, 53, 32 and 54 related to central, eastern, southern and western Africa, respectively. The countries with the most datapoints were Nigeria (20), Kenya (14), South Africa (12) and Ghana and Ethiopia (11 each). In total, 110 datapoints (75.9%) represented the estimated incidence of catastrophic health expenditure at the population level, whereas 35 (24.1%) represented the disease-specific incidence. Most datapoints (98.6%; 143/145) came from cross-sectional studies and were nationally representative (68.3%; 99/145). The sample size of the studies ranged from 87 to 73 329 households (median: 4165; interquartile range: 8379).

**Table 1:** Studies included, meta-analysis of catastrophic health expenditure in sub-Saharan Africa, 2000–2021

Study	Study country	Study design	Data source and year	Study population	No. of households	No. of households with catastrophic health expenditure^a^		AXIS score^b^
						Greater than 10% of total household expenditure	Greater than 40% of household non-food expenditure	
Adesina & Ogaji 2020^22^	Nigeria	Cross-sectional	Primary data from a cross-sectional household survey, 2017	Community	525	173	67	15
Adisa 2015^23^	Nigeria	Cross-sectional	Nigeria General Household and Population Survey, 2010	Households in the community with adults aged ≥ 50 years	1 176	113	ND	16
Aidam et al. 2016^24^	Ghana	Cross-sectional	Primary data from a cross-sectional household survey, 2013	Community	117	ND	38	11
Ajayi et al. 2021^25^	Nigeria	Cross-sectional	Primary data from a cross-sectional household survey, 2018	Community	971	153	53	13
Akalu et al. 2012^26^	Ethiopia	Cross-sectional	Primary data from a cross-sectional household survey, 2007	Households in the community with recent use of reproductive health services	1 015	ND	619	10
Akazili et al. 2017^27^	Ghana	Cross-sectional	Ghana Living Standard Survey, 2005/2006	Community	8 687	455	229	15
Akinkugbe et al. 2012^28^	Botswana and Lesotho	Cross-sectional	Botswana Household and Expenditure Survey, 2002/2003, and Lesotho Household Budget Survey, 2002/2003	Community	6 053 (Botswana); 6 882 (Lesotho)	ND	450 (Botswana); 86 (Lesotho)	13
Aregbesola & Khan 2018^29^	Nigeria	Cross-sectional	Harmonised Nigeria Living Standard Survey, 2009/2010	Community	38 700	6347	5302	15
Arsenault et al. 2013^30^	Mali	Case–control	Project data on maternal mortality in the Kayes region, 2008–2011	Households in the community with recent use of reproductive health services	484	162	ND	14
Aryeetey et al. 2016^31^	Ghana	Cross-sectional	Primary data from a cross-sectional household survey, 2009	Community	3 300	ND	891	15
Asante et al. 2007^32^	Ghana	Cross-sectional	Primary data from a population-based cross-sectional household survey, 2005	Households in the community with recent use of reproductive health services	2 250	236	ND	9
Assebe et al. 2020^33^	Ethiopia	Cross-sectional	Ethiopia Health Account and cross-sectional health facility-based survey for tuberculosis, 2016/2017	Households in the community containing an individual with an HIV infection or tuberculosis	1 006 (HIV); 787 (tuberculosis)	197 (HIV); 315 (tuberculosis)	ND	18
Ataguba 2012^34^	Nigeria	Cross-sectional	Nigerian National Living Standard Survey, 2003/2004	Community	19 518	4606	ND	10
Atake & Amendah 2018^35^	Togo	Cross-sectional	Primary data from a population-based cross-sectional household survey, 2016	Community	1 180	390	115	17
Attia-Konan et al. 2019^36^	Côte d’Ivoire	Cross-sectional	Côte d’Ivoire National household living standards survey, 2015	Community	12 899	ND	519	12
Babikir et al. 2018^37^	South Africa	Panel survey	National Income Dynamics Study, 2013	Community	10 236	ND	1372	15
Bandoh 2016^38^	Ghana	Cross-sectional	Ghana Living Standards Survey (round 6), 2012	Community	16 772	2573	75	15
Barasa et al. 2017^39^	Kenya	Cross-sectional	Kenya Household Expenditure and Utilization Survey, 2013	Community	33 675	ND	2216	15
Beaulière et al. 2010^40^	Côte d’Ivoire	Cross-sectional	Primary data from a population-based cross-sectional survey, 2007	Households in the community with an HIV patient	1 190	ND	143	15
Bermudez-Tamayo et al. 2017^41^	Mali	Case–control	Primary data from a population-based cross-sectional survey, 2015	Households in the community with a diabetes mellitus patient	993	332	ND	14
Bonfrer et al. 2017^42^	Kenya	Cross-sectional	Primary data from a population-based cross-sectional household survey, 2011	Community	1 226	ND	37	14
Borde et al. 2020^43^	Ethiopia	Cross-sectional	Primary data from a population-based and community-based cohort study, 2017	Households in the community with recent use of reproductive health services	794	362	91	20
Brinda et al. 2014^44^	United Republic of Tanzania	Cross-sectional	United Republic of Tanzania National Panel Survey, 2008/2009	Community	3 265	ND	588	14
Buigut et al. 2015^45^	Kenya	Cross-sectional	Kenya Indicator Development for Surveillance of Urban Emergencies project, 2011	Community	8 171	1863	ND	15
Castillo-Riquelme et al. 2008^46^	Mozambique and South Africa	Cross-sectional	Primary data from a population-based cross-sectional household survey, 2001/2002	Community	828 (Mozambique); 827 (South Africa)	351 (Mozambique); 64 (South Africa)	324 (Mozambique); 68 (South Africa)	12
Chansa et al. 2018^47^	Zambia	Cross-sectional	Zambia Living Conditions Monitoring Survey, 2010, and Zambia Household Health Expenditure and Utilization Survey, 2014	Community	20 000 (2010); 12 260 (2014)	ND	768 (2010); 220 (2014)	16
Chuma et al. 2012^48^	Kenya	Cross-sectional	Kenya Ministry of Health national survey, 2007	Community	8 414	1481	2137	12
Chuma et al. 2007^49^	Kenya	Cross-sectional	Primary data from a cross-sectional household survey, 2004	Community	1 924	227	ND	12
Cleary et al. 2013^50^	South Africa	Cross-sectional	Primary data from a population-based cross-sectional survey, 2011	Households in the community with an HIV or tuberculosis patient or with recent use of reproductive health services	1 267 (HIV); 1 229 (tuberculosis); 1 231 (reproductive health service use)	288 (HIV); 406 (tuberculosis); 814 (reproductive health service use)	ND	18
Counts & Skordis-Worrall 2016^51^	United Republic of Tanzania	Panel survey	Kagera Health and Development Surveys, 1991–2010	Community	900	ND	179	14
Dickerson et al. 2020^52^	Malawi	Cross-sectional	Malawi Integrated Household Surveys, 2004 and 2010	Community	11 271	ND	516	14
Doamba et al. 2013^53^	Burkina Faso	Cross-sectional	Burkina Faso Enquête Intégrale sur les Conditions de Vie des Ménages, 2009	Community	8 404	ND	121	10
Ebaidalla 2021^54^	Sudan	Cross-sectional	Sudan National Baseline Household Surveys, 2009 and 2014	Community	7 913 (2009); 11 953 (2014)	4 036 (2009); 6 455 (2014)	ND	10
Edoka et al. 2017^55^	Sierra Leone	Cross-sectional	Sierra Leone Integrated Household Surveys, 2003 and 2011	Community	6 800 (2003); 3 700 (2011)	3 407 (2003); 1 184 (2011)	ND	16
Ekirapa-Kiracho et al. 2021^56^	Uganda	Cross-sectional	Primary data from a population-based cross-sectional survey, 2015	Households in the community with a child aged < 5 years with pneumonia	693	478	270	18
Etiaba et al. 2016^57^	Nigeria	Cross-sectional	Primary data from a population-based cross-sectional survey, 2013	Households in the community with an HIV patient	1 557	ND	171	15
Fink et al. 2013^58^	Burkina Faso	Pre-intervention baseline survey	Nouna Health and Demographic Surveillance System survey, 2003	Community	983	82	ND	16
Frimpong et al. 2021^59^	Ghana	Cross-sectional	Ghana Living Standards Survey (round 6), 2013	Community	9 395	ND	1847	16
Gabani & Guinness 2019^60^	Liberia	Cross-sectional	Liberia Household Income and Expenditure Survey, 2014	Community	4 085	74	74	17
Gunda et al. 2017^61^	Zimbabwe	Cross-sectional	Primary data from a cross-sectional household survey, 2015	Community	109	ND	38	11
Hailemichael et al. 2019^62^	Ethiopia	Case–control	Primary data from a cross-sectional household survey, 2015	Community	257	42	ND	16
Hailemichael et al. 2019^63^	Ethiopia	Case–control	Primary data from a cross-sectional household survey, 2015	Community	579	104	146	16
Harris et al. 2011^64^	South Africa	Cross-sectional survey	South Africa National Household Survey, 2008	Community	4 668	490	ND	14
Hassen 2019^65^	Mauritania	Cross-sectional survey	Permanent Household Living Conditions Survey, 2014	Community	9 557	1081	370	18
Hilaire 2018^66^	Benin	Cross-sectional survey	Benin Integrated Modular Survey on Living Conditions of Households, 2009	Community	15 411	1540	ND	16
Ibukun & Komolafe 2018^67^	Nigeria	Cross-sectional	Nigeria General Household Survey, 2015/2016	Community	4 581	ND	1649	10
Ichoku et al. 2009^68^	Nigeria	Cross-sectional	Primary data from a cross-sectional household survey, 2004	Community	1 497	326	ND	11
Ilesanmi et al. 2014^69^	Nigeria	Cross-sectional	Primary data from a cross-sectional household survey, 2012	Community	714	ND	47	11
Janssens et al. 2016^70^	Nigeria	Cross-sectional	Primary data from a cross-sectional household survey, 2012	Community	1 450	ND	128	14
Kaonga et al. 2019^71^	Zambia	Cross-sectional	Zambian Household Health Expenditure and Utilization Survey, 2014	Community	12 000	1368	ND	13
Khatry et al. 2013^72^	Mauritania	Cross-sectional	Enquête Permanente sur les Conditions de Vie des ménages, 2008	Community	13 705	ND	566	10
Kihaule 2015^73^	United Republic of Tanzania	Cross-sectional survey	United Republic of Tanzania Demographic and Health Survey, 2009	Community	10 300	ND	1922	10
Kihaule et al. 2019^74^	United Republic of Tanzania	Case–control	Primary data from a population-based cross-sectional household survey, 2018	Community	1 080	ND	420	9
Kimani & Maina 2015^75^	Kenya	Cross-sectional	Kenya Household Health Expenditure and Utilization Survey, 2003	Community	8 844	593	911	16
Kimani et al. 2016^76^	Kenya	Cross-sectional	Kenya Household Expenditure and Utilization Survey, 2007	Community	8 844	1269	988	8
Kiros et al. 2020^77^	Ethiopia	Cross-sectional	Ethiopia Household Consumption and Expenditure and Welfare Monitoring Survey, 2015/2016	Community	30 229	635	ND	14
Kirubi et al. 2021^78^	Kenya	Cross-sectional	Kenya National Tuberculosis Programme Patient Cost Survey, 2017	Households in the community with a tuberculosis patient	1 071	171	ND	19
Koch & Setshegetso 2020^79^	South Africa	Cross-sectional	South African Income and Expenditure Surveys, 2000, 2005/2006 and 2010/2011	Community	22 437 (2000); 20 994 (2005); 25 119 (2010)	980 (2000); 2438 (2005); 2505 (2010)	254 (2000); 570 (2005); 499 (2010)	13
Kusi et al. 2015^80^	Ghana	Cross-sectional	Primary data from a population-based cross-sectional household survey, 2011	Community	2 430	ND	87	13
Kwesiga et al. 2020^81^	Uganda	Cross-sectional	Uganda National Household Surveys, 2005/2006, 2009/2010, 2012/2013 and 2016/2017	Community	7 400 (2005); 6 887 (2009); 7 500 (2012); 17 320 (2016)	1658 (2005); 1474 (2009); 1035 (2012); 2459 (2016)	ND	11
Laisin et al. 2020^82^	Cameroon	Cross-sectional	Cameroon Household Consumption Survey IV, 2014	Community	10 303	6698	ND	7
Lamiraud et al. 2005^83^	South Africa	Cross-sectional	World Health Survey, 2002	Community	2 602	ND	273	11
Laokri et al. 2018^84^	Democratic Republic of the Congo	Pre-intervention baseline survey	Primary data from a population-based cross-sectional survey, 2014	Community	4 120	700	ND	12
Liu et al. 2019^85^	Rwanda	Cross-sectional	Rwanda Integrated Living Conditions Surveys, 2014 and 2016	Community	14 125 (2014); 14 548 (2016)	ND	254 (2014); 669 (2016)	15
Lu et al. 2012^86^	Rwanda	Cross-sectional	Rwanda Integrated Living Conditions Survey, 2000	Community	6 408	ND	763	13
Lu et al. 2017^87^	Rwanda	Cross-sectional	Rwanda Integrated Living Conditions Surveys, 2005 and 2010	Community	6900 (2005); 14 308 (2010)	ND	511 (2005); 1173 (2010)	14
Macha 2015^88^	United Republic of Tanzania	Cross-sectional	Primary data from a population-based cross-sectional household survey, 2014	Community	274	73	ND	10
Masiye et al. 2016^89^	Zambia	Cross-sectional	Zambia Household Health Expenditure and Utilization Survey, 2014	Community	11 847	1327	1102	15
Mills et al. 2012^90^	United Republic of Tanzania	Cross-sectional	United Republic of Tanzania Household Budget Survey, 2000	Community	22 178	ND	346	16
Mulaga et al. 2021^91^	Malawi	Cross-sectional	Malawi Integrated Household Survey, 2016/2017	Community	12 447	515	167	18
Angèle et al. 2021^92^	Democratic Republic of the Congo	Cross-sectional	Primary data from a population-based cross-sectional survey, 2015	Households in the community with recent use of reproductive health services	411	167	ND	17
Mussa 2016^93^	Malawi	Cross-sectional	Malawi Third Integrated Household Survey, 2010/2011	Community	12 271	304	117	17
Muttamba et al. 2020^94^	Uganda	Cross-sectional	Primary data from a cross-sectional household survey, 2015	Households in the community with a tuberculosis patient	1 178	71	ND	16
Mwai & Muriithi 2016^95^	Kenya	Cross-sectional	Kenya Household Expenditure Survey, 2007	Community	8 453	ND	1449	9
Nabyonga et al. 2013^96^	Uganda	Cross-sectional	Uganda National Household Survey, 2002	Community	9 711	ND	3322	12
Nannini et al. 2021^97^	Uganda	Pre-intervention baseline survey	Primary data from a population-based cross-sectional household survey, 2019	Community	320	ND	52	16
Negin et al. 2017^98^	South Africa	Cross-sectional	Study on global AGEing and adult health (SAGE), South Africa Wave 1, 2007/2008	Households in the community with adults aged ≥ 50 years	2 969	ND	192	17
Ngcamphalala & Ataguba 2018^99^	Eswatini	Cross-sectional	Swaziland Household Income and Expenditure Survey, 2009/2010	Community	3 167	307	86	16
Nguyen et al. 2011^100^	Ghana	Cross-sectional	Primary data from a cross-sectional household survey, 2019	Community	2 500	51	25	16
Njagi et al. 2020^101^	Kenya	Cross-sectional survey	Kenya Household Expenditure and Utilization Survey, 2007	Community	3 728	ND	425	13
Njuguna et al. 2017^102^	Kenya	Cross-sectional	Kenya Household Health Utilization and Expenditure Survey, 2013	Community	33 675	ND	2122	9
Ntambue et al. 2019^103^	Democratic Republic of the Congo	Mixed-methods	Primary data from a population-based cross-sectional survey, 2015	Households in the community with recent use of reproductive health services	1 627	ND	261	19
Nundoochan et al. 2019^104^	Mauritius	Cross-sectional	Mauritius Household Budget Surveys, 2001/2002, 2006/2007 and 2012	Community	6 720 (2001); 6 720 (2006); 6 720 (2012)	388 (2001); 438 (2006); 595 (2012)	41 (2001); 62 (2006); 84 (2012)	16
Nyakangi 2020^105^	Kenya	Cross-sectional	Kenya Household Health Utilization and Expenditure Survey, 2018	Households in the community with a patient with a chronic noncommunicable disease	37 500	ND	2985	13
Obembe et al. 2021^106^	Nigeria	Cross-sectional	Primary data from a population-based cross-sectional survey, 2017	Households in the community with a patient who had recent surgery	450	280	ND	19
Obse & Ataguba 2020^107^	Ethiopia	Cross-sectional	Ethiopian Household Consumption Expenditure Survey, 2010/2011	Community	28 032	1144	230	12
Ogaji & Adesina 2018^108^	Nigeria	Cross-sectional	Primary data from a population-based cross-sectional household survey, 2012	Community	525	172	ND	13
Olasehinde & Olaniyan 2017^109^	Nigeria	Cross-sectional	Harmonized Nigeria Living Standard Survey, 2010	Community	73 329	ND	4180	13
Olutumise et al. 2021^110^	Nigeria	Cross-sectional	Primary data from a population-based cross-sectional household survey, 2019	Community	427	268	ND	12
Onah & Govender 2014^111^	Nigeria	Cross-sectional survey	Primary data from a cross-sectional household survey, 2010	Community	411	44	ND	14
Onoka et al. 2011^112^	Nigeria	Cross-sectional	Primary data from a cross-sectional household survey, 2008	Community	1 128	ND	167	11
Onwujekwe et al. 2012^113^	Nigeria	Cross-sectional	Primary data from a population-based cross-sectional household survey, 2008	Community	3 070	ND	881	7
Onwujekwe et al. 2012^114^	Nigeria	Cross-sectional	Primary data from a cross-sectional household survey, 2011	Community	4 873	ND	1229	11
Onwujekwe et al. 2016^115^	Nigeria	Cross-sectional	Primary data from a cross-sectional household survey, 2013	Community	1 409	568	108	19
Opara et al. 2021^116^	Uganda	Cross-sectional	Primary data from a population-based cross-sectional survey, 2018	Households in the community with a rheumatic heart disease patient	87	35	ND	17
Pedrazzoli et al. 2018^117^	Ghana	Cross-sectional	Primary data from a population-based cross-sectional survey, 2016	Households in the community with a tuberculosis patient	691	509	ND	13
Saksena et al. 2010^118^	Burkina Faso, Chad, Côte d’Ivoire, Democratic Republic of the Congo, Eswatini, Ethiopia, Ghana, Kenya, Malawi, Mali, Mauritania Mauritius, Namibia, Zambia and Zimbabwe	Cross-sectional	WHO World Health Survey, 2002–2003	Community	4 948 (Burkina Faso); 4 875 (Chad); 3 245 (Côte d’Ivoire); 3 070 (Democratic Republic of the Congo); 3 121 (Eswatini); 5 090 (Ethiopia); 4 165 (Ghana); 4 640 (Kenya); 5 551 (Malawi); 5 209 (Mali); 3 907 (Mauritania); 3 958 (Mauritius); 4 379 (Namibia); 6 165 (Zambia); 4 264 (Zimbabwe)	ND	1000 (Burkina Faso); 593 (Chad); 569 (Côte d’Ivoire); 672 (Democratic Republic of the Congo); 299 (Eswatini); 485 (Ethiopia); 708 (Ghana); 457 (Kenya); 397 (Malawi); 997 (Mali); 478 (Mauritania); 325 (Mauritius); 175 (Namibia); 283 (Zambia); 307 (Zimbabwe)	15
Salari et al. 2018^119^	Kenya	Cross-sectional	Kenya Household Health Utilization and Expenditure Survey, 2018	Community	37 500	4013	2663	12
Sanoussi & Ametoglo 2019^120^	Togo	Cross-sectional	Questionnaire of Basic Indicators of Well Being survey, 2015	Community	2 400	504	168	12
Scheil-Adlung et al. 2006^121^	Kenya, Senegal and South Africa	Cross-sectional	Kenya Household Expenditure and Utilization Survey (Kenya), 2003, and WHO World Health Survey (Senegal and South Africa), 2003	Community	4 354 (Kenya); 3 259 (Senegal); 2 579 (South Africa)	ND	186 (Kenya); 686 (Senegal); 308 (South Africa)	15
Séne & Cissé 2015^122^	Senegal	Cross-sectional	Senegal Poverty Monitoring Survey, 2011	Community	5 953	372	ND	10
Shikuro et al. 2020^123^	Ethiopia	Cross-sectional	Primary data from a cross-sectional household survey, 2017	Community	479	ND	108	18
Sichone 2020^124^	Zambia	Cross-sectional	Zambia Household Health Expenditure & Utilization Survey, 2014	Households in the community with a child aged < 5 years with malaria	2 164	355	ND	13
Sow et al. 2013^125^	Senegal	Cross-sectional	Enquêtes de Suivi de la Pauvreté au Sénégal, 2011	Community	18 000	ND	467	10
Su et al. 2006^126^	Burkina Faso	Cross-sectional	Nouna Health District Household Survey, 2000/2001	Community	774	ND	67	10
Tolla et al. 2017^127^	Ethiopia	Cross-sectional	Primary data from a population-based cross-sectional survey, 2017	Households in the community with a cardiovascular disease patient	589	158	ND	18
Ujunwa et al. 2014^128^	Nigeria	Cross-sectional	Primary data from a cross-sectional household survey, 2012	Community	809	ND	281	10
Van Duinen et al. 2021^129^	Sierra Leone	Cross-sectional	Primary data from a population-based cross-sectional survey, 2017	Households in the community with a woman who has undergone a caesarean section	1 146	138	ND	17
Wang et al. 2016^130^	Malawi	Cross-sectional	Primary data from a population-based cross-sectional survey, 2012	Households in the community with a chronic noncommunicable disease patient	1 199	ND	321	15
Xu et al. 2006^131^	Uganda	Cross-sectional	Uganda Socio-economic Surveys, 2000 and 2003	Community	10 691 (2000); 9 710 (2003)	ND	337 (2000); 284 (2003)	13
Zeng et al. 2018^132^	Zimbabwe	Cross-sectional	Zimbabwe National Statistics Agency Household Survey, 2016	Community	7 135	899	ND	13

AXIS: appraisal tool for cross-sectional studies; HIV: human immunodeficiency virus; ND: not determined; WHO: World Health Organization.

^a^ The threshold for catastrophic health expenditure was either 10% of total household expenditure or 40% of household non-food expenditure.

^b^ Study quality was assessed using the AXIS tool:^17^ an AXIS score of 0–10 indicated a high risk of bias and a score of 11–20 indicated a low risk.

The quality of 95 of the 111 included studies (85.6%) was rated as high (AXIS score: 11–20), whereas the quality of the remaining 16 (14.4%) was rated as low (AXIS score: 0–10). When the risk of bias was weighted according to each study’s sample size, studies covering 88.6% (921 704/1 040 620) of households included were rated as having a low risk of bias, whereas those covering 11.4% (118 916/1 040 620) were judged to have some quality concerns or were rated as having a high risk of bias. Of note, all studies included used sample frames and sampling techniques that closely represented the underlying population (as assessed using AXIS tool items 5 and 6).

### Household expenditure threshold

**Fig. 3:**
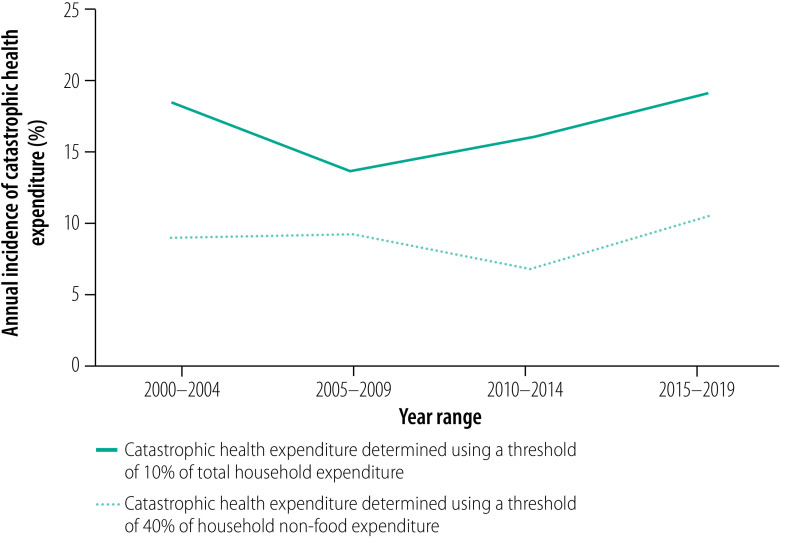
Trends in the incidence of catastrophic health expenditure in sub-Saharan Africa, 2000–2019

When the threshold for catastrophic health expenditure was defined as 10% of total household expenditure, the pooled annual incidence across 50 datapoints, which covered 462 151 households, was 16.5% (95% CI: 12.9–20.4; Table 2). Further details are available in the data repository.^133^ In the sensitivity analyses, excluding the 10% of studies with the smallest sample sizes yielded a slightly lower pooled incidence of 15.0% (95% CI: 11.4–19.0; 45 datapoints; 459 989 households), whereas excluding the 10% of studies with the largest sample sizes yielded a slightly higher pooled incidence of 17.8% (95% CI: 13.8–22.3; 45 datapoints; 317 634 households). The difference was not great. When poor-quality studies were excluded, the estimated pooled incidence was 15.4% (95% CI: 12.2–19.0; 46 datapoints; 441 233 households). Between 2000 and 2019, the pooled incidence initially declined but increased between 2005–2009 and 2015–2019 (Fig. 3). 

**Table 2:** Characteristics of subgroups of studies that defined catastrophic health expenditure as 10% of total household expenditure, sub-Saharan Africa, 2000–2021

Study subgroup definition	No. of countries in subgroup	No. of incidence datapoints in subgroup (%)	No. of households in subgroup (%)	Study sample size, range	Pooled incidence of catastrophic health expenditure^a^, % (95% CI)	Between-study heterogeneity, *I*^2^ %
**All studies**	22	50 (100)	462 151 (100)	274–38 700	16.5 (12.9–20.4)	99.9
**Study period**						
2000–2009	11	21 (42.0)	209 028 (45.2)	983–38 700	15.6 (11.1–20.7)	99.9
2010–2019	19	29 (58.0)	253 123 (54.8)	274–30 229	17.1 (11.9–23.1)	99.9
**Sub-Saharan African region^b^**						
Central	2	2 (4.0)	14 423 (3.1)	4120–10 303	50.6 (49.8–51.4)	NA
Eastern	6	17 (34.0)	173 865 (37.6)	274–30 229	16.0 (9.4–23.9)	99.8
Southern	5	10 (20.0)	132 085 (28.6)	3167–25 119	8.4 (6.0–11.1)	99.7
Western	9	21 (42.0)	141 778 (30.7)	411–38 700	19.6 (14.8–24.9)	99.8
**Country income status^c^**						
Low	10	18 (36.0)	175 523 (38.0)	983–30 229	22.0 (12.4–33.5)	99.9
Lower middle	10	25 (50.0)	193 250 (41.8)	274–38 700	15.4 (12.9–18.0)	99.6
Upper middle	2	7 (14.0)	93 378 (20.2)	4668–25 119	8.0 (5.8–10.6)	99.4
**Social health insurance coverage**						
< 10%	22	48 (96.0)	438 659 (94.9)	274–38 700	16.7 (12.9–20.8)	99.9
≥ 10%	2	2 (4.0)	23 492 (5.1)	6720–16 772	13.3 (12.9–13.8)	NA
**UHC service coverage index**						
< 45	15	30 (60.0)	258 021 (55.8)	274–38 700	22.0 (15.6–29.1)	99.9
≥ 45	8	20 (40.0)	204 130 (44.2)	1924–25 119	9.6 (7.6–11.8)	99.6
**Data source**						
Primary	4	9 (18.0)	11 250 (2.4)	274–4 120	22.7 (12.8–34.3)	99.4
Secondary	20	41 (82.0)	450 901 (97.6)	983–38 700	15.3 (11.5–19.5)	99.9
**Sample size**						
< 1000 households	3	7 (14.0)	4 116 (0.9)	411–983	31.3 (19.0–45.2)	98.8
≥ 1000 households	20	43 (86.0)	458 035 (99.1)	1176–38 700	14.5 (10.9–18.5)	99.9
**Study design**						
Observational	21	49 (98.0)	461 168 (99.8)	274–38 700	16.0 (12.5–19.9)	99.9
Pre-interventional	1	1 (2.0)	983 (0.2)	NA	45.3 (42.2–48.4)	NA
**Representativeness of study sample**						
Regionally representative	6	12 (24.0)	19 388 (4.2)	274–8 171	24.7 (16.3–34.2)	99.5
Nationally representative	20	38 (76.0)	442 763 (95.8)	1176–38 700	14.2 (10.4–18.5)	99.9
**Publication status**						
Not peer reviewed	5	5 (10.0)	65 605 (14.2)	2400–28 032	10.9 (5.8–17.5)	99.8
Peer reviewed	21	45 (90.0)	396 546 (85.8)	274–38 700	17.2 (13.2–21.6)	99.9
**Study quality^d^**						
Low risk of bias	20	46 (92.0)	441 233 (95.5)	411–38 700	15.4 (12.2–19.0)	99.9
High risk of bias	4	4 (8.0)	20 918 (4.5)	274–10 303	30.8 (5.7–64.8)	99.9

CI: confidence interval; NA: not applicable; UHC: universal health coverage.

^a^ The threshold for catastrophic health expenditure was defined as 10% of total household expenditure.

^b^ Countries in sub-Saharan Africa were grouped into four regions using the African Union classification.^10^

^c^ Countries’ income status was classified as low, lower middle or upper middle using the World Bank’s classification.^9^

^d^ Study quality was assessed using the appraisal tool for cross-sectional studies (AXIS) score:^17^ an AXIS score of 0–10 indicated a high risk of bias and a score of 11–20 indicated a low risk.

At the country level, Cameroon and Sudan had the highest and second highest incidence, at 65.0% (95% CI: 64.1–65.9) and 52.8% (95% CI: 52.1–53.5), respectively (details available in the data repository).^133^ Regionally, the pooled incidence for countries in central and western Africa was higher than that for the whole of sub-Saharan Africa (Table 2). The incidence was highest for countries in central Africa, at 50.6% (95% CI: 49.8–51.4; two datapoints; 14 423 households), and lowest for countries in southern Africa, at 8.4% (95% CI: 6.0–11.1; 10 datapoints; 132 085 households). Univariate meta-regression analysis indicated that the between-study variation in the pooled incidence was associated with: (i) study quality as assessed using the AXIS score (*P*-value 0.005); (ii) the country’s income status (*P*-value  0.005); and (iii) the country’s UHC service coverage index (*P*-value 0.005). Full details are available in the data repository.^133^ However, multivariable meta-regression analysis indicated that no variable was independently associated with between-study differences in the estimated pooled incidence.

### Non-food expenditure threshold

When the threshold for catastrophic health expenditure was defined as 40% of household non-food expenditure, the pooled annual incidence across 84 datapoints, which covered 795 355 households, was 8.7% (95% CI: 7.2–10.3; Table 3). Further details are available in the data repository.^133^ In the sensitivity analyses, excluding the 10% of studies with the smallest sample sizes yielded a slightly lower pooled incidence of 7.9% (95% CI: 6.5–9.5; 75 datapoints; 789 746 households), whereas excluding the 10% of studies with the largest sample sizes yielded a slightly higher pooled incidence of 9.3% (95% CI: 7.5–11.3; 75 datapoints; 480 710 households). The incidence estimates were similar. When poor-quality studies were excluded, the pooled incidence was slightly lower at 7.9% (95% CI: 6.4–9.5; 73 datapoints; 691 778 households). Between 2000 and 2019, the pooled incidence initially decreased but increased between 2010–2014 and 2015–2019 (Fig. 3).

**Table 3:** Characteristics of subgroups of studies that defined catastrophic health expenditure as 40% of household non-food expenditure, sub-Saharan Africa, 2000–2021

Study subgroup definition	No. of countries in subgroup	No. of incidence datapoints in subgroup (%)	No. of households in subgroup (%)	Study sample size, range	Pooled incidence of catastrophic health expenditure^a^, % (95% CI)	Between-study heterogeneity, *I*^2^ %
**All studies**	25	84 (100)	795 355 (100)	117–73 329	8.7 (7.2–10.3)	99.8
**Study period**						
2000–2009	23	47 (56.0)	341 950 (43.0)	774–38 700	9.2 (6.9–11.7)	99.8
2010–2019	16	37 (44.0)	453 405 (57.0)	117–73 329	8.1 (6.3–10.0)	99.8
**Sub-Saharan African region^b^**						
Central	2	2 (2.4)	7 945 (1.0)	3070–4 875	15.6 (14.9–16.5)	NA
Eastern	6	30 (35.7)	325 837 (41.0)	320–37 500	8.9 (6.5–11.7)	99.9
Southern	8	19 (22.6)	192 374 (24.2)	2579–25 119	4.7 (3.2–6.4)	99.7
Western	9	33 (39.3)	269 199 (33.8)	117–73 329	10.8 (8.0–14.0)	99.8
**Country income status^c^**						
Low	9	23 (27.4)	182 466 (22.9)	320–28 032	7.6 (4.8–11.1)	99.8
Lower middle	11	48 (57.1)	487 490 (61.3)	117–73 329	10.8 (8.8–13.0)	99.8
Upper middle	5	13 (15.5)	125 399 (15.8)	2579–25 119	4.1 (2.3–6.3)	99.7
**Social health insurance coverage**						
< 10%	25	76 (90.5)	730 022 (91.8)	320–73 329	9.0 (7.5–10.7)	99.8
≥ 10%	3	8 (9.5)	65 333 (8.2)	117–16 772	5.7 (2.0–11.1)	99.8
**UHC service coverage index**						
< 45	13	37 (44.0)	331 666 (41.7)	479–73 329	11.7 (8.7–15.1)	99.9
≥ 45	14	47 (56.0)	463 689 (58.3)	117–37 500	6.6 (5.2–8.2)	99.8
**Data source**						
Primary	6	16 (19.0)	24 316 (3.1)	117–4 873	15.5 (9.3–23.1)	98.5
Secondary	25	68 (81.0)	771 039 (96.9)	900–73 329	7.4 (6.0–8.9)	99.8
**Sample size**						
< 1000 households	6	9 (10.7)	5 609 (0.7)	117–971	16.4 (9.9–24.1)	98.1
≥ 1000 households	25	75 (89.3)	789 746 (99.3)	1080–73 329	7.9 (6.5–9.5)	99.8
**Study design**						
Observational	25	83 (98.8)	795 035 (99.9)	117–73 329	8.6 (7.2–10.2)	99.8
Pre-interventional	1	1 (1.2)	320 (0.1)	NA	16.2 (12.6–20.6)	NA
**Representativeness of study sample**						
Regionally representative	7	18 (21.4)	26 396 (3.3)	117–4 873	15.4 (9.7–22.2)	99.5
Nationally representative	25	66 (78.6)	768 959 (96.7)	2400–73 329	7.2 (5.8–8.8)	99.8
**Publication status**						
Not peer reviewed	8	11 (13.1)	110 659 (13.9)	2400–28 032	5.7 (3.1–9.0)	99.8
Peer reviewed	25	73 (86.9)	684 696 (86.1)	117–73 329	9.2 (7.6–10.9)	99.8
**Study quality^d^**						
Low risk of bias	25	73 (86.9)	691 778 (87.0)	117–73 329	7.9 (6.4–9.5)	99.8
High risk of bias	6	11 (13.1)	103 577 (13.0)	774–33 675	14.7 (8.9–21.7)	99.9

CI: confidence interval; NA: not applicable; UHC: universal health coverage.

^a^ The threshold for catastrophic health expenditure was defined as 40% of household non-food expenditure.

^b^ Countries in sub-Saharan Africa were grouped into four regions using the African Union classification.^10^

^c^ Countries’ income status was classified as low, lower middle or upper middle using the World Bank’s classification.^9^

^d^ Study quality was assessed using the appraisal tool for cross-sectional studies (AXIS) score:^17^ an AXIS score of 0–10 indicated a high risk of bias and a score of 11–20 indicated a low risk.

At the country level, the Democratic Republic of the Congo and Mali had the highest and second highest incidence, at 21.9% (95% CI: 20.5–23.4) and 19.1% (95% CI: 18.1–20.2), respectively (details in the data repository).^133^ Regionally, the estimated pooled incidence for countries in central, eastern and western Africa were all higher than the pooled incidence for the whole of sub-Saharan Africa (Table 3). The pooled incidence for lower-middle-income countries was higher, at 10.8% (95% CI: 8.8–13.0; 48 datapoints; 487 490 households), than for low-income countries, at 7.6% (95% CI: 4.8–11.1; 23 datapoints; 182 466 households). Univariate meta-regression analysis indicated that the between-study variation in pooled incidence was associated with: (i) whether primary or secondary data had been used (*P*-value < 0.001); (ii) study quality as assessed using the AXIS score (*P*-value < 0.001); (iii) the country’s income status (*P*-value 0.001); and (iv) the country’s UHC service coverage index (*P*-value 0.001). Full details are available in the data repository.^133^ However, multivariable meta-regression analysis indicated that only study data type (*P*-value 0.024) and study quality (*P*-value 0.009) were independently associated with between-study differences in estimated pooled incidence. On average, studies that used secondary data reported a lower incidence of catastrophic health expenditure than those using primary data.

### Disease-specific catastrophic expenditure 

Estimates of the pooled incidence of catastrophic health expenditure for different disease groups (Table 4) were generally higher than estimates for the whole population (Table 2 and Table 3).

**Table 4:** Characteristics of disease-specific subgroups of studies, meta-analysis of catastrophic health expenditure in sub-Saharan Africa, 2000–2021

Catastrophic health expenditure threshold and study subgroup	No. of countries in subgroup	No. of incidence datapoints in subgroup	No. of households in subgroup	Study sample size, range	Pooled incidence of catastrophic health expenditure^a^, % (95% CI)	Between-study heterogeneity, *I*^2^ %
**10% of total household expenditure**						
Noncommunicable diseases	3	5	2 505	87–993	26.0 (18.7–34.1)	94.3
Maternal, neonatal and child health	7	7	6 766	411–2 250	37.2 (18.4–58.2)	99.6
Emergency obstetric surgery	5	5	3 431	120–1 231	55.9 (26.5–83.2)	99.7
HIV/AIDS and tuberculosis	6	8	8 638	691–1 409	29.9 (17.4–44.2)	99.5
HIV/AIDS	3	3	3 682	1006–1 409	27.1 (15.6–40.5)	98.7
Tuberculosis	6	6	6 365	691–1 409	33.0 (16.1–52.7)	99.6
Acute childhood illnesses	4	4	4 512	693–2 164	31.6 (9.9–58.8)	99.7
**40% of household non-food expenditure**						
Noncommunicable diseases	4	5	49 151	579–37 500	11.8 (6.9–17.8)	99.4
Maternal, neonatal and child health	2	3	3 436	794–1 627	27.5 (4.8–59.5)	99.7
Emergency obstetric surgery	1	2	317	120–197	67.6 (62.3–72.7)	NA
HIV/AIDS and tuberculosis	4	5	18 396	1190–11 271	8.1 (5.4–11.3)	94.0
HIV/AIDS	4	5	18 396	1190–11 271	8.2 (5.0–12.1)	99.7
Tuberculosis	1	1	1 409	NA	7.7 (6.4–9.2)	NA
Acute childhood illnesses	4	4	2 457	109–828	28.7 (12.0–49.6)	99.1

CI: confidence interval; HIV/AIDS: human immunodeficiency virus/acquired immunodeficiency syndrome; NA: not applicable.

^a^ The threshold for catastrophic health expenditure was defined as 10% of total household expenditure or 40% of household non-food expenditure, as indicated.

### Publication bias

For the population-level meta-analyses, visual inspection of funnel plots suggested there was no publication bias. However, Egger’s test for small-study effects gave a significant result (*P*-value 0.003 when the threshold was 10% of total household expenditure and *P*-value < 0.001 when it was 40% of household non-food expenditure). We were unable to determine whether the small-study effect was driven by publication bias because there was substantial heterogeneity in the data. For both thresholds, trim-and-fill analysis suggested that publication bias was absent (details available in the data repository).^133^ Similar assessments performed for the disease-specific meta-analyses also suggested that publication bias was absent.

### Evidence quality 

The quality of the evidence used for estimating the incidence of catastrophic health expenditure at the population level with both thresholds was graded as high as there was no serious risk of bias, imprecision, indirectness or inconsistency (Table 5) . However, the quality of the evidence used for estimating the incidence of disease-specific catastrophic expenditure varied from low to high because, for some disease groups, there was serious imprecision, a serious risk of bias and serious inconsistency across the studies.

**Table 5:** Evidence quality, by study subgroup, meta-analysis of catastrophic health expenditure, sub-Saharan Africa, 2000–2021

Meta-analysis outcome	No. of households in analysis	Evidence quality criterion^a^				GRADE evidence quality^b^
		Risk of bias^c^	Imprecision^d^	Indirectness^e^	Inconsistency^f^	
**Incidence of catastrophic health expenditure in community studies**						
With a threshold of 10% of total household expenditure	462 151	Not serious	Not serious	Not serious	Not serious	High
With a threshold of 40% of household non-food expenditure	795 355	Not serious	Not serious	Not serious	Not serious	High
**Incidence of catastrophic health expenditure in studies of specific disease groups**						
Noncommunicable diseases						
With a threshold of 10% of total household expenditure	1 669	Not serious	Serious	Not serious	Serious	Low
With a threshold of 40% of household non-food expenditure	48 572	Not serious	Not serious	Not serious	Serious	Moderate
Maternal, neonatal and child health						
With a threshold of 10% of total household expenditure	6 766	Not serious	Not serious	Not serious	Serious	Moderate
With a threshold of 40% of household non-food expenditure	3 436	Serious	Not serious	Not serious	Serious	Low
HIV/AIDS and tuberculosis						
With a threshold of 10% of total household expenditure	8 638	Not serious	Not serious	Not serious	Serious	Moderate
With a threshold of 40% of household non-food expenditure	18 396	Not serious	Not serious	Not serious	Not serious	High
Acute childhood illnesses						
With a threshold of 10% of total household expenditure	4 512	Not serious	Not serious	Not serious	Serious	Moderate
With a threshold of 40% of household non-food expenditure	2 457	Not serious	Not serious	Not serious	Serious	Moderate

GRADE: Grading of Recommendations, Assessment, Development and Evaluation; HIV/AIDS: human immunodeficiency virus/acquired immunodeficiency syndrome.

^a^ The quality of the evidence was assessed using the Grading of Recommendations, Assessment, Development and Evaluation (GRADE) approach.^20^

^b^ The GRADE evidence quality refers to the systematic and explicit consideration of study design, study quality, consistency and directness of evidence in judgements.

^c^ There was a serious risk of bias if ≥ 25% of studies had a risk of bias (i.e. an inappropriate sampling method or statistical analysis).

^d^ There was imprecision if ≥ 25% of studies had a small sample size.

^e^ There was indirectness if≥ 25% of studies did not use valid and reliable methods of data collection.

^f^ There was inconsistency if there was heterogeneity between the studies (i.e. the difference between the upper and lower limits of the 95% confidence interval was ≥ 10%).

## Discussion

Our findings suggest that one in six households in sub-Saharan Africa experienced a financial catastrophe when seeking health care between 2000 and 2019. Our review also indicates that the incidence of catastrophic health expenditure increased between 2010–2014 and 2015–2019. This increase could be due to the higher cost of health care, of both medications and medical consultations.^15^^,^^134^^,^^135^ The result is financial difficulty for households, and exerts fiscal pressure on the strained health budget of many countries.^134^

Over the last two decades, rapid population growth, ageing, urbanization and a sedentary lifestyle have increased the incidence of noncommunicable diseases in sub-Saharan Africa.^136^ Catastrophic health expenditure is unlikely to fall in the near future unless drastic measures are taken to counter this rise.^137^ In addition, the coronavirus disease 2019 pandemic affected livelihoods and reduced household incomes, thereby further exposing households to medical impoverishment.^138^

The incidence of catastrophic health expenditure we found in sub-Saharan Africa was lower than in China in the last decade,^139^ but higher than in Europe,^140^^–^^142^ Asia,^134^^,^^143^^,^^144^ and South America,^145^^,^^146^ irrespective of the definition used. The incidence may be higher than in Europe and South America because of slow progress in developing a health financing system in sub-Saharan Africa that encourages risk pooling and prepayment contributions and because of continuing overreliance on out-of-pocket payments.^147^^,^^148^

The high incidence of catastrophic health expenditure we found for specific diseases suggests that health-care costs are driven not just by the cost of treatment for acute, life-threatening health shocks, such as emergency surgery or intensive care, but also by the relatively small – but recurrent – cost of chronic illness. We found that about a quarter of households affected by a noncommunicable disease incurred catastrophic health-care costs (when defined as 10% of total household expenditure), a substantially higher figure than for the general population. This result is consistent with growing evidence that noncommunicable disease is a major driver of health-care costs for households.^137^^,^^149^^–^^151^ In sub-Saharan Africa, the rising burden of noncommunicable diseases has not been matched by measures to curb health-care costs. Policies that simultaneously tackle these diseases and protect households affected by them are urgently needed if the region is to achieve SDG 3.4.1 (i.e. to reduce premature deaths from noncommunicable disease by 25% by 2025) or 1.1.1 (to eradicate extreme poverty).^152^

Most sub-Saharan African countries are also burdened by epidemics of infectious diseases, including human immunodeficiency virus/acquired immunodeficiency syndrome (HIV/AIDS), tuberculosis, malaria and pneumonia.^136^ We found that the incidence of catastrophic health expenditure was generally higher among households with a patient with HIV/AIDS or tuberculosis than in the rest of the population. This finding suggests that, despite out-of-pocket payment exemptions for people with these conditions, affected households still experience catastrophic health expenditure. The reason could be the high cost of treatment before diagnosis (e.g. from inappropriate care-seeking or irrational drug use), lost income due to prolonged hospitalization, or non-medical expenditure (e.g. for travel or nutritional supplements).^33^^,^^153^ Because the rapid expansion of free antiretroviral therapy and tuberculosis treatment has helped increase life expectancy, financial protection must be extended beyond exemptions for out-of-pocket payments for direct treatment costs.

Our study also showed that the incidence of catastrophic health expenditure was high among people using maternal, neonatal and child health care services. Vulnerable families in most sub-Saharan African countries who require health care for severe obstetric complications, neonatal admission, or paediatric hospitalization or surgery are particularly at risk.^154^ The sub-Saharan African region alone accounts for two thirds of maternal deaths globally each year.^155^ Substantial progress in reducing maternal, neonatal and child mortality is unlikely before countries act to protect households from catastrophic out-of-pocket expenditure when accessing maternal, neonatal and child health-care services.^92^^,^^103^ The elimination of user fees, for example, could increase access to these services while shielding households from impoverishment.^103^

Our study has several strengths. The study is a methodological improvement on previous studies as we used several measures of catastrophic health expenditure.^134^^,^^139^^,^^143^^,^^144^ As payment for health care can crowd out both food and non-food expenditure, it was important to examine health expenditures using the two thresholds of 10% of total household expenditure and 40% of household non-food expenditure. Also, as we included only population-based studies, our findings are more generalizable to the whole population than those of previous studies.

There are also some limitations. First, survey-based evaluations of catastrophic health expenditure understate the risk faced by poorer households that are unable to seek care because of costs and thus report zero health expenditure. Consequently, our estimates should be taken as lower bounds of the true incidence of catastrophic health expenditure in sub-Saharan Africa. Second, in the absence of a universal definition, we defined catastrophic health expenditure using the thresholds of 10% of total household expenditure and 40% of non-food expenditure, as did 96% of eligible studies. A different definition could have given different pooled incidences. Finally, information on the UHC service coverage index was available only for 2015 and 2017 and data on social insurance coverage were sparse,^12^^,^^13^ which limited confidence in findings related to those two variables.

Despite these limitations, our study provides important evidence for discussions on policy and health financing reform. By demonstrating that a substantial portion of the sub-Saharan African population experience catastrophic costs when accessing health care, our study underscores the urgency of designing effective and inclusive social protection mechanisms. Although identifying interventions was not a study objective, our findings highlight the need for measures such as insurance premium exceptions, co-payment exceptions, free medications and free diagnostic tests for households at most risk. Developing a social insurance system is the preferred long-term solution to catastrophic health expenditure and impoverishment in the region. In the short-term, increased donor funding for both public health care services and country-specific social safety nets are needed to ensure access for poor people. In addition, country-specific, targeted programmes can help reduce health inequity. Regular, nationally representative surveys remain critical tools for tracking health expenditure and for identifying the individuals, households and disease populations most at risk.

The catastrophic health expenses experienced by many people in sub-Saharan Africa threaten poverty alleviation efforts. Stronger financial protection is critically needed in the region if continued progress is to be made towards achieving UHC and meeting the attendant SDGs.
